# Is network meta-analysis as valid as standard pairwise meta-analysis? It all depends on the distribution of effect modifiers

**DOI:** 10.1186/1741-7015-11-159

**Published:** 2013-07-04

**Authors:** Jeroen P Jansen, Huseyin Naci

**Affiliations:** 1 Mapi, 180 Canal Street, Suite 503, Boston, MA 02114, USA; 2 Tufts University School of Medicine, 145 Harrison Avenue, Boston, MA 02111, USA; 3 LSE Health & Social Care, London School of Economics & Political Science, Cowdray House, 20 Houghton Street, London WC2A 2AE, UK

**Keywords:** Bias, Comparative effectiveness, Confounding, Effect modification, Indirect comparison, Meta-analysis, Mixed treatment comparison, Network meta-analysis, Randomized controlled trial, Systematic review

## Abstract

**Background:**

In the last decade, network meta-analysis of randomized controlled trials has been introduced as an extension of pairwise meta-analysis. The advantage of network meta-analysis over standard pairwise meta-analysis is that it facilitates indirect comparisons of multiple interventions that have not been studied in a head-to-head fashion. Although assumptions underlying pairwise meta-analyses are well understood, those concerning network meta-analyses are perceived to be more complex and prone to misinterpretation.

**Discussion:**

In this paper, we aim to provide a basic explanation when network meta-analysis is as valid as pairwise meta-analysis. We focus on the primary role of effect modifiers, which are study and patient characteristics associated with treatment effects. Because network meta-analysis includes different trials comparing different interventions, the distribution of effect modifiers cannot only vary across studies for a particular comparison (as with standard pairwise meta-analysis, causing heterogeneity), but also between comparisons (causing inconsistency). If there is an imbalance in the distribution of effect modifiers between different types of direct comparisons, the related indirect comparisons will be biased. If it can be assumed that this is not the case, network meta-analysis is as valid as pairwise meta-analysis.

**Summary:**

The validity of network meta-analysis is based on the underlying assumption that there is no imbalance in the distribution of effect modifiers across the different types of direct treatment comparisons, regardless of the structure of the evidence network.

## Background

Randomized controlled trials (RCTs) are considered as the gold standard of whether a health intervention works and/or whether it is better than another. Although often placed at the top of evidence hierarchies, single RCTs rarely provide adequate information for addressing the evidence demands of patients, clinicians and policymakers. Instead, each trial provides a piece of evidence that, when taken together with others, addresses important questions for patients, clinicians, and other healthcare decision-makers [1]. Traditional pairwise meta-analyses of RCTs are increasingly used to synthesize the results of different trials evaluating the same intervention(s) to obtain an overall estimate of the treatment effect of one intervention relative to the control.

In the last decade, network meta-analysis has been introduced as a generalization of pairwise meta-analysis. When the available RCTs of interest do not all compare the same interventions but each trial compares only a subset of the interventions of interest, it is possible to develop a network of RCTs where all trials have at least one intervention in common with another. Such a network allows for indirect comparisons of interventions not studied in a head-to-head fashion [2]. For example, the treatment effects from trials comparing treatments B relative to A (AB trials) and trials comparing treatments C relative to A (AC trials) can be pooled to obtain an indirect estimate for the comparison between treatments B and C [3-5]. Even when a trial comparing treatments C and B (BC trial) exists, combining the direct estimates with the results of indirect comparisons can result in refined estimates as a broader evidence base is considered [6,7]. In general, if the available evidence base consists of a network of interlinked multiple RCTs involving treatments compared directly, indirectly, or both, the entire body of evidence can be synthesized by means of network meta-analysis [8].

Although assumptions underlying standard pairwise meta-analyses of direct comparisons are well understood, those concerning network meta-analysis for both direct and indirect comparisons might be perceived to be more complex, and might be prone to misinterpretation [9-11]. In this paper, we aim to compare pairwise meta-analysis with network meta-analysis with a specific focus on the primary role of effect modifiers as the common cause of heterogeneity and bias. We discuss effect modification first within individual trials, then in standard pairwise meta-analyses of multiple randomized trials, and finally in network meta-analyses.

## Discussion

### Effect modification and within-study variation of treatment effects

**Figure 1:**
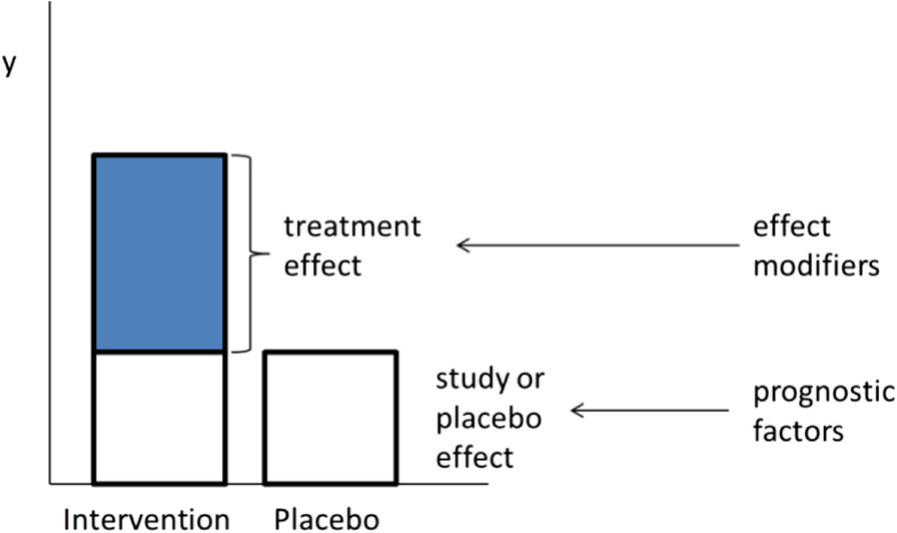
Treatment vs study effect in a randomized controlled trial, and the role of effect modifiers.

As a result of randomly allocating patients to the intervention or control group, well-designed RCTs achieve high internal validity by balancing (un)known and (un)measured prognostic factors across intervention groups within a trial (Figure 1). By doing so, the difference in observed outcomes in randomized trials, defined as the treatment effect (or effect size), can be attributed to differences in interventions compared (assuming that there are no other systematic differences resulting in bias, such as issues in concealment of treatment allocation) [12].

Within an RCT, different groups of participants can respond to treatments differently. Hence, it is possible to have subgroups of participants with different treatment effects. This variation in true treatment effects is called heterogeneity and is caused by differences in patient characteristics within a trial that are effect modifiers (Figure 1) [13]. When heterogeneity occurs within an individual trial, it is referred to as within-study heterogeneity. Within-study heterogeneity occurs particularly in trials without strict entry criteria. For instance, RCTs evaluating the efficacy of cholesterol-lowering statins often include a mixture of patients with and without a history of coronary artery disease. As these subgroups of patients respond to statin therapy differently (that is, individuals with a history of coronary artery disease tend to derive greater relative mortality reduction as patients without a history of coronary artery disease), disease history is an effect modifier and results in within-study heterogeneity of treatment effects.

### Effect modification and between-study variation of treatment effects

**Figure 2:**
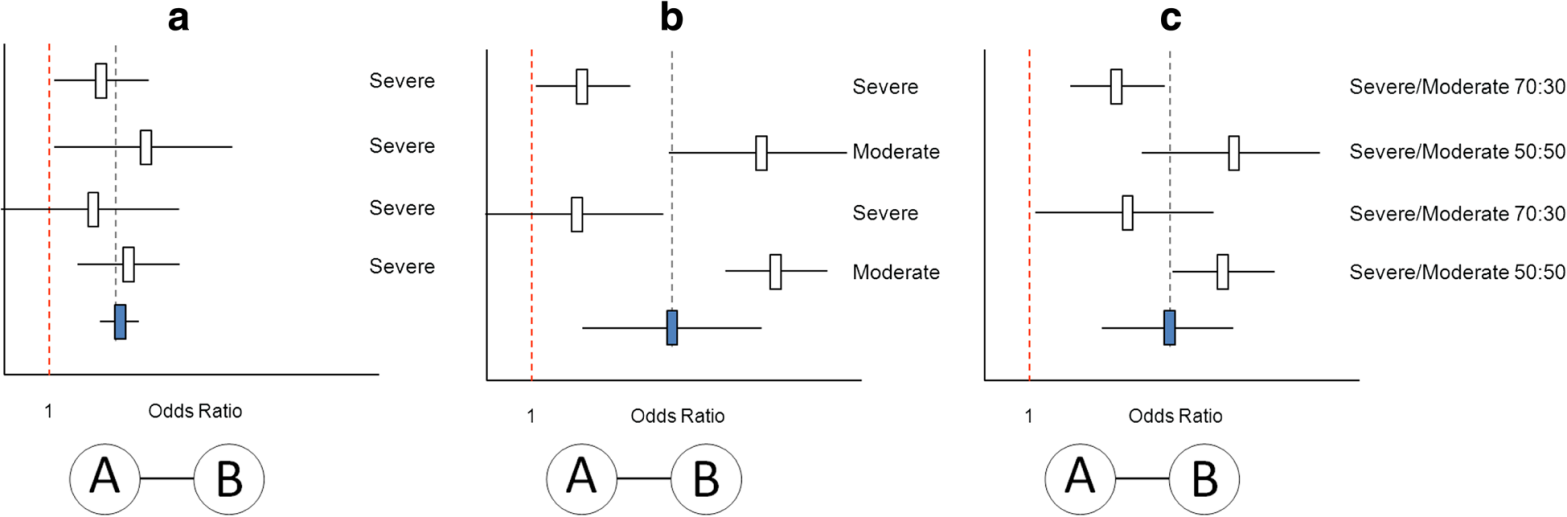
**Standard pairwise meta-analysis of four AB trials (comparing treatment B relative to A). (a)** No differences in the effect modifier ‘disease severity’ across studies and therefore no between-study heterogeneity. **(b)** Extreme differences in the effect modifier across studies and therefore between-study heterogeneity. (Not useful to pool the studies). **(c)** Differences in the distribution of the effect modifier across studies and therefore between-study heterogeneity. Given the inclusion of both severe and moderate patients in each of the studies there is also within-study heterogeneity, but this cannot be observed without access to subgroup or individual patient-level data.

With standard pairwise meta-analysis, treatment effects of different studies comparing the same interventions are combined using statistical techniques to obtain a ‘pooled’ treatment effect estimate [14]. Although randomization is maintained within trials, it does not hold across the set of trials included in the meta-analysis; patients are not randomized to different trials. As a result, there are situations where there are systematic differences in study characteristics or the distribution of patient characteristics across trials. If these characteristics are effect modifiers (that is, influence the treatment effects), then there are systematic differences in treatment effects across trials: between-study heterogeneity. Hence, in a standard meta-analysis there can be both within-study heterogeneity and between-study heterogeneity (Figure 2). If meta-analyses are based on the reported treatment effects for the overall trial population in publications, there is typically no information on the within-study heterogeneity (which would be available in individual patient-level data).

Pooling different studies in the presence of extreme between-study heterogeneity does not introduce bias, but may render the results of the meta-analysis irrelevant [15]. When considering the scenario presented in Figure 2b, the pooled result is not applicable to a moderate-only population or a severe-only population. In this situation, an alternative approach would be to perform separate meta-analyses for the studies with severe and moderate populations. In Figure 2c a more realistic scenario is presented where there is both within-study and between-study variation in the distribution of the effect modifier. Given the use of published treatment effects we can only observe between-study heterogeneity in treatment effects. Combining the results of these four heterogeneous studies is in essence similar to the pooling of the treatment effect across subgroups of one trial characterized by different values of the effect modifier.

### Effect modification and between-comparison variation of treatment effects

In a standard pairwise meta-analysis where each trial compares the same interventions with the same control (say only AB studies) the only source of variation in the treatment effects between trials can be due to the presence of effect modifiers that are different from one trial to the next: between-study heterogeneity. In a network meta-analysis, studies concern different treatment comparisons (for example, AB studies, AC studies). Hence, there is an additional source of variability of treatment effects between trials, which is the treatment comparison itself. In a network meta-analysis or indirect comparison of RCTs there can be three types of variation of treatment effects: (1) true within-study variation of treatment effects (which is only observable with individual patient-level data or reporting of subgroups), (2) true between-study variation in treatment effects for a particular treatment comparison, and (3) true between-comparison variation in treatment effects.

**Figure 3:**
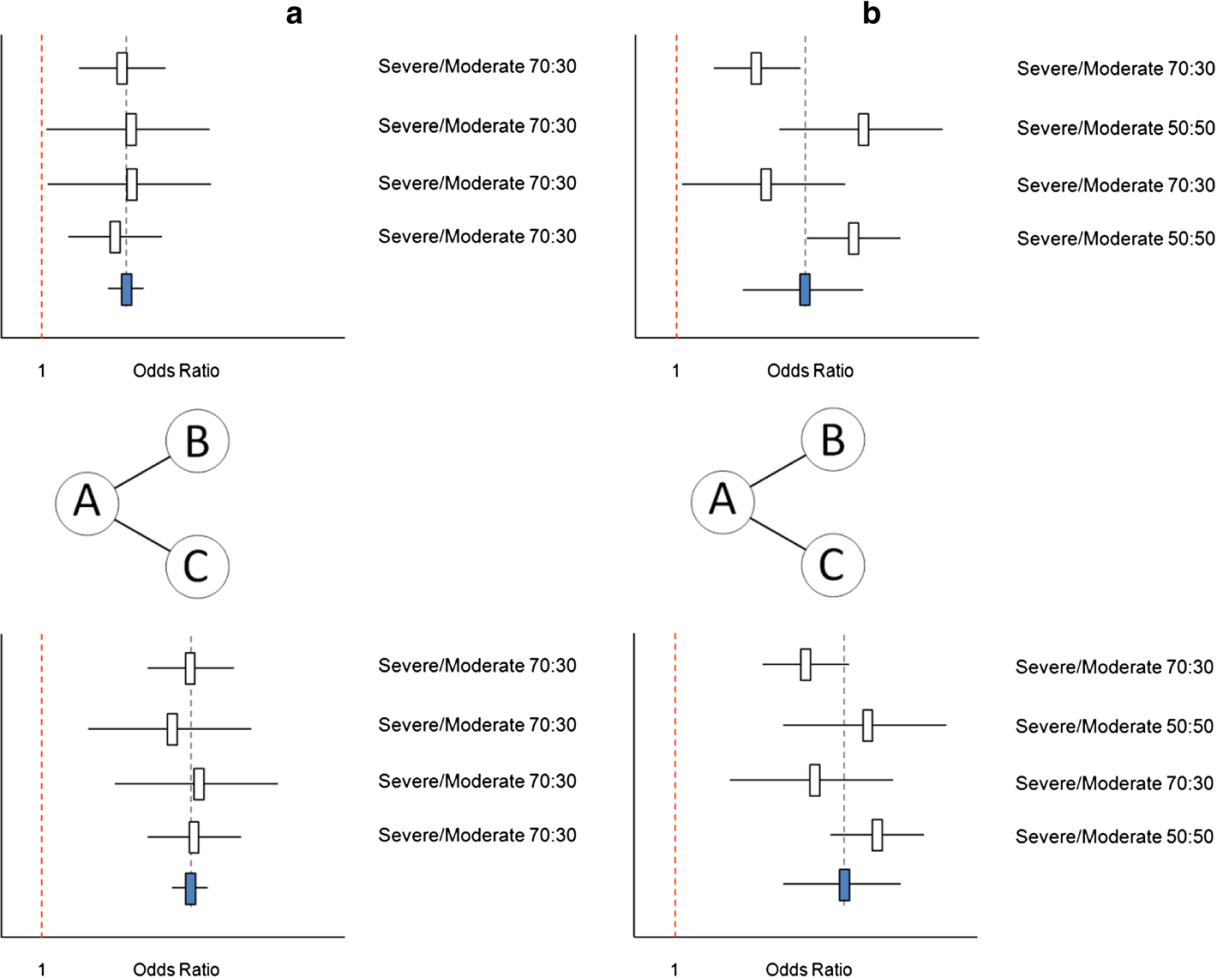
**Valid network meta-analysis of AB trials (comparing treatment B relative to A) and AC trials (comparing treatment C relative to A). (a)** Forest plot of four AB studies and four AC studies. There are no differences in the effect modifier across studies within comparisons and no imbalance in the distribution of the effect modifier between comparisons. Hence, there is no heterogeneity, and no bias in the indirect comparison estimate of C vs B. **(b)** Forest plot of four AB studies and four AC studies. There are differences in the effect modifier across studies within comparisons, but no imbalance in the distribution of the effect modifier between comparisons. Hence, there is heterogeneity, but no bias in the indirect comparison estimate of C vs B.

Figure 3 shows two scenarios where the findings of the network meta-analysis are as valid as in a standard pairwise meta-analysis. In Figure 3a a network meta-analysis of four AB and four AC studies is presented. For the AB studies there is no variation in the distribution of disease severity, which is an effect modifier, and therefore no heterogeneity in AB treatment effects. (For this example we assume that severity is the only effect modifier and it is known and measured.) For the four AC studies there is also no variation in the effect modifier, and consequently, no between-study heterogeneity. Furthermore, the distribution of the effect modifier for the AB comparison is the same as for the AC comparison (that is, no between-comparison variation). As such, any observed difference in the pooled treatment effect between the AB and AC studies can be attributable to the difference between the two types of comparisons: intervention B or C. In this scenario, the indirect estimate for the BC treatment effect is unbiased.

In Figure 3b a network meta-analysis is presented with variation in the distribution of the effect modifier across the AB studies resulting in between-study heterogeneity. The same is observed for the AC comparison. Since the distribution of severity across the four AB studies is the same as for the four AC studies, the difference between the pooled estimates of AB and AC is only due to the actual difference in the interventions compared. The indirect estimate for the BC comparison is again unbiased.

**Figure 4:**
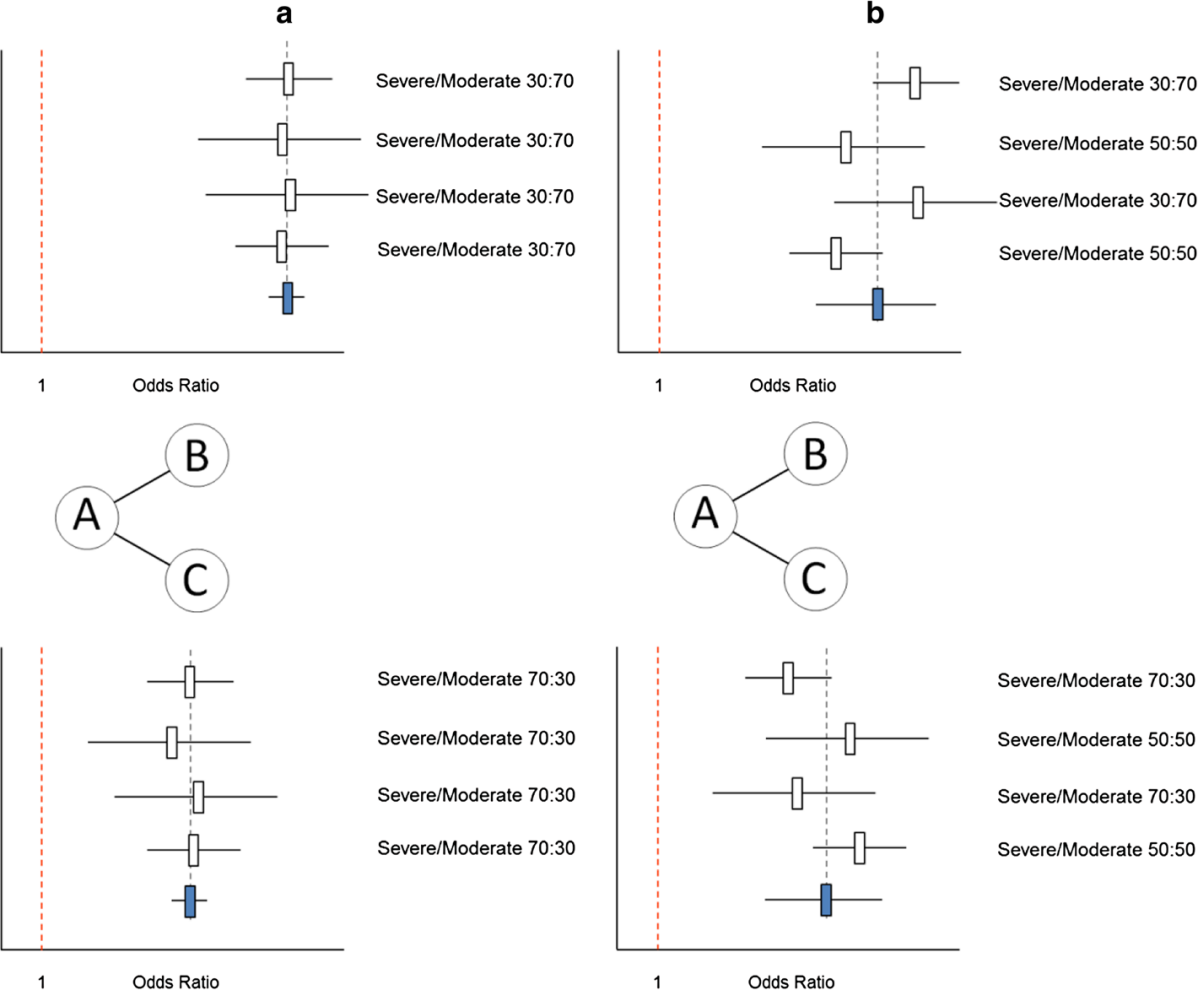
**Biased network meta-analysis of AB trials (comparing treatment B relative to A) and AC trials (comparing treatment C relative to A). (a)** Forest plot of four AB studies and four AC studies. There are no differences in the effect modifier across studies within comparisons, but an imbalance in the distribution of the effect modifier between comparisons. Hence, there is no between-study heterogeneity, but a biased indirect comparison estimate of C vs B. **(b)** Forest plot of four AB studies and four AC studies. There are differences in the distribution of the effect modifier across studies within comparisons, as well as an imbalance in the distribution of the effect modifier between comparisons. Hence, there is between-study heterogeneity, and a biased indirect comparison estimate of C vs B.

Figure 4 shows two scenarios where the findings of the network meta-analyses are biased. In the scenario presented in Figure 4a, all AB and AC comparisons are free of heterogeneity. However, there is variation in the distribution of the effect modifier between the comparisons. For the AB studies there is a 30:70 distribution of patients with severe and moderate disease, while the opposite is the case for the AC studies. Given this imbalance in the effect modifier between comparisons the observed difference between the pooled treatment effects of the AB studies and the AC studies cannot be considered to be only attributable to the difference in interventions. The indirect comparison is affected by confounding bias due to the imbalance in the effect modifier [16]. In a similar fashion as in an observational study where confounding bias arises from a common cause of the exposure and the disease, in indirect comparisons of RCTs confounding bias occurs when a covariate has an impact on the treatment effect (that is, effect modifier) and is also associated with the type of treatment comparison. From a counterfactual perspective one can state that an indirect comparison of an AB and AC study is affected by confounding bias if the population of the AB study presents an imperfect substitute of what the target population in the AC study would have been like under the counterfactual condition that intervention B was provided instead of intervention C and therefore the indirect treatment effect of C versus B is different than the causal effect of C relative to B.

A similar scenario is presented in Figure 4b. Here, there is an imbalance (or between-comparison variation) in the distribution of the effect modifier. In addition, there is heterogeneity across the AB studies as well as the AC studies due to variation in the effect modifier between studies within the comparisons. This variation results in biased indirect comparison estimates.

An imbalance in the distribution of effect modifiers across the different comparisons, sometimes referred to as a violation of the similarity or consistency assumptions [17], results in a violation of transitivity. Transitivity means that if C is more efficacious than B, and B is more efficacious than A, then C has to be more efficacious than A. It is important to acknowledge that there is always the risk of unknown imbalances in effect modifiers and accordingly the risk of residual confounding bias, even if all observed effect modifiers are balanced. However, this does not imply that network meta-analyses are as prone to bias as observational studies. In non-randomized comparative studies the relative treatment effect between the two interventions are affected by confounding bias if either the prognostic factors of the study effects or modifiers of treatment effects are not balanced across the intervention groups. Given the randomized nature of the individual trials included in network meta-analyses, and we only compare treatment effects of interventions that are part of the same network of RCTs, we only have to worry about the effect modifiers as a source of confounding bias.

### Heterogeneity and inconsistency as the two sides of the same effect-modification coin

**Figure 5:**
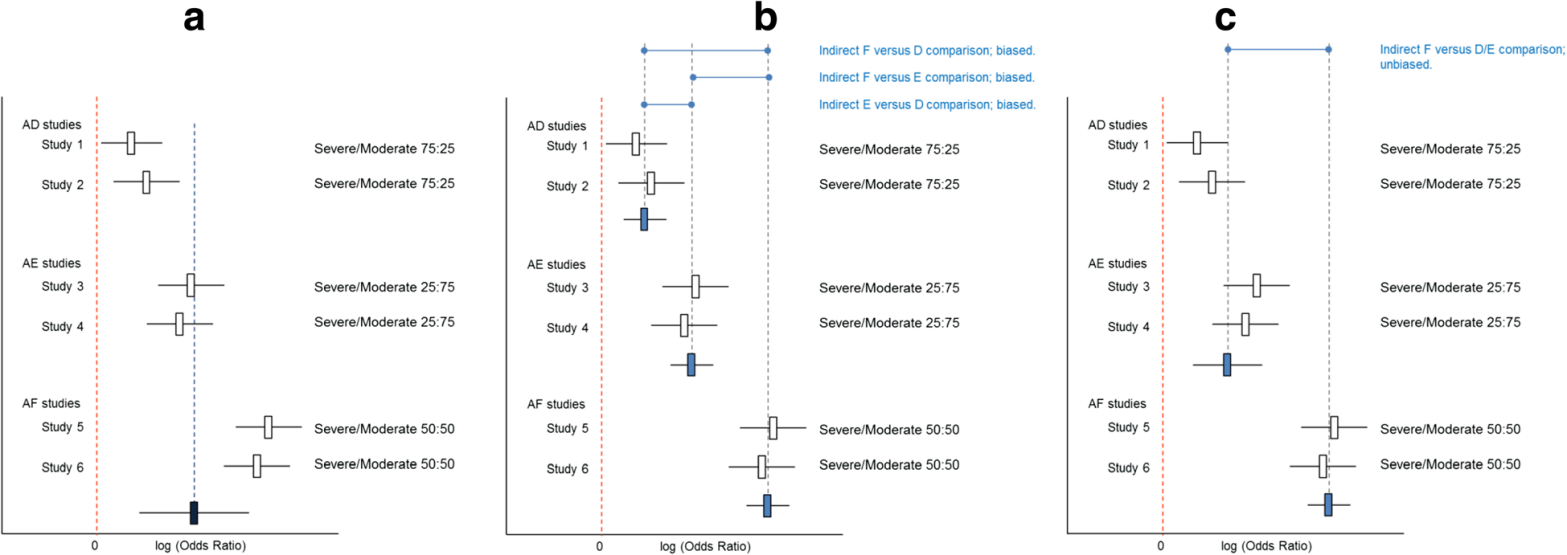
**Given a certain distribution of effect modifiers across studies, grouping of studies for the analysis has an impact on heterogeneity and inconsistency. (a)** Pooled results of all trials as obtained with a pairwise meta-analysis results in great amount of heterogeneity. **(b)** Network meta-analysis with grouping of studies according to type of comparison (AD, AE, or AF) results in inconsistency in the absence of heterogeneity. **(c)** Network meta-analysis with grouping of studies by treatment class (AD and AE vs AF) results in absence of inconsistency but presence of between-study heterogeneity (across studies 1 to 4).

As explained above, between-study heterogeneity results from the variation in effect modifiers within comparisons, whereas inconsistency results from the imbalance in effect modifiers between comparisons. Figure 5a presents the results of six hypothetical studies where each study evaluates a biological treatment (that is, D, E, and F) relative to placebo (A) for rheumatoid arthritis. A pairwise meta-analysis of the six studies provides an average treatment effect for the efficacy of biological treatment relative to placebo. Given the different biologics and differences in the distribution of the effect modifier (that is, disease severity) there is fair amount of heterogeneity. In Figure 5b the results of a network meta-analysis are presented with pooled results for each of the three biologics relative to placebo. Although there is no heterogeneity for the AD comparison, AE comparison, or AF comparison, there is an imbalance in the distribution of the effect modifiers and the indirect comparisons are biased. If treatments D and E are from the same class of biologics (for example, infliximab and adalimumab are both tumor necrosis factor (TNF)α blockers), and we assume that their treatment effects are exchangeable, we can pool studies one through four and compare the efficacy of this class of biologics with the efficacy of biological agent F. This network meta-analysis is presented in Figure 5c. Now, there is no imbalance in the average distribution of the effect modifier across the two types of comparisons (that is, D and E vs A, and F vs A); for both comparisons the distribution of moderate and severe patients is 50:50. However, there is between-study heterogeneity across studies one through four. This heterogeneity is smaller than the overall heterogeneity across all six studies. Based on this hypothetical illustration we can infer the following: Given a distribution of effect modifiers across a range of studies in a network of RCTs, the distribution within and between comparisons is determined by the grouping of these studies for the analysis. Please note that with this hypothetical example we are not promoting the pooling of different treatments in order to get a similar overall distribution of effect modifiers for the different direct comparisons involved in the network meta-analysis. Grouping of treatments should primarily be determined by the research question of interest. The example was just presented to illustrate that heterogeneity and inconsistency can be considered two sides of the same coin.

If a network meta-analysis consists of an evidence base where for some interventions there is both direct and indirect evidence, inconsistency can be evaluated by comparing the treatment effect estimates obtained from the direct comparison, with those obtained from the indirect comparisons for the same contrast [18-21]. For example, in a network of RCTs that consists of AB, AC, and BC studies, inconsistency can be evaluated by comparing the direct comparison BC with the indirect estimate for BC obtained from the AB and AC studies. For comparisons where only indirect evidence is available, say the BC comparison in a network of only AB and AC studies, inconsistency cannot be assessed this way, and can only be explored by comparing the average distribution of effect modifiers between AB and AC studies [22].

In network meta-analysis, consistency is sometimes referred to as a separate assumption from the similarity assumption suggesting that the similarity assumption relates to indirect comparisons, and the consistency assumption only applies to situations where there is both direct and indirect evidence for a certain treatment comparison [17]. However, portraying similarity and consistency as separate assumptions is not very useful given the fact that any valid network meta-analysis is based on the assumption that there is no imbalance in the distribution of effect modifiers across the different types of treatment comparisons (i.e. transitivity), regardless of the structure of the evidence network.

### Practical implications

In an attempt to bridge the gap between the conceptual considerations and realities of performing network meta-analysis, a brief discussion on practical implications is warranted. Frequently, there are several observed differences in trial and patient characteristics across the different direct comparisons. Deciding which covariates are effect modifiers based on observed differences in results across trials can be challenging and potentially lead to false conclusions regarding the sources of inconsistency [22]. We recommend that researchers first generate a list of potential treatment effect modifiers for the interventions of interest based on prior knowledge or subgroup results of individual studies before comparing results between studies. Next, the distribution of study and patient characteristics that are determined to be likely effect modifiers should be compared across studies to identify any potential imbalances between different types of direct comparisons [16].

If there are a sufficient number of studies included in the network meta-analysis, it may be possible to perform a meta-regression analysis where the treatment effect of each study is not only a function of the treatment comparison of that study but also related to an effect modifier [23]. This allows indirect comparisons with adjustment for confounding bias due to differences in the measured effect modifiers between studies if the estimated relationship between effect modifier and treatment effect is not greatly affected by bias [23,24]. Network meta-analysis is typically based on study-level data extracted from published reports of trials. Adjusting for imbalances in patient level effect modifiers based on study-level data can be prone to ecological bias [24-26]. Having access to patient level data (at least for a subset of studies) can improve parameter estimation of network meta-analysis models with adjustment for differences in patient-level covariates across comparisons. Hence, it is recommended to use patient-level data where available [25,26].

Even in cases where relative treatment effect modifiers are identified in advance, practical challenges remain in regards to their availability in published reports thereby limiting meta-regression analysis [9]. Nevertheless, we recommend network meta-analysis reports to include a discussion of known effect modifiers, their availability in the published body of evidence, and how their distribution across studies may affect the findings.

## Summary

Network meta-analysis is different from pairwise meta-analysis in the sense that there is not only one type of treatment comparison, but multiple treatment comparisons. As a result, the distribution of effect modifiers cannot only vary across studies for a particular comparison (as with pairwise meta-analysis), but also between comparisons. If there is an imbalance in the distribution of effect modifiers between different types of comparisons, indirect comparisons will be biased and the validity of the network meta-analysis is compromised. In the Additional file 1 this key requirement for transitivity is also demonstrated with mathematical equations. If the assumption that there are no imbalances in effect modifiers between different types of direct comparisons can be defended or seems appropriate given the available RCTs then network meta-analysis is as valid as pairwise meta-analysis. If there are sources of bias that affect the direct comparisons of the individual studies (for example, information bias, publication bias, or selective outcome reporting bias) then the pooled results of both pairwise meta-analysis and network meta-analysis are affected. However, when indirect evidence can wash out trial-specific biases that are sometimes not identifiable in a head-to-head meta-analysis, indirect estimates obtained with a network meta-analysis might be preferable [19,20]. Network meta-analysis has the advantage that it allows for indirect comparisons, more data are incorporated in the analysis, and the bigger picture is tackled, while a single pairwise meta-analysis offers a very fragmented picture.

## Competing interests

This manuscript was written without specific funding. The authors declare that they have no competing interests.

## Authors’ contributions

JPJ and HN are both responsible for the development of the content and writing of the manuscript. Both authors read and approved the final manuscript.

## Pre-publication history

The pre-publication history for this paper can be accessed here:

http://www.biomedcentral.com/1741-7015/11/159/prepub

## Supplementary Material

Additional file 1 Equations to illustrate that the imbalance in the distribution of effect modifiers across different types of direct comparisons violates the consistency assumption of network meta-analysis and result in biased indirect estimates. Click here for file
