# Supplementary material for: Is network meta-analysis as valid as standard pairwise meta-analysis? It all depends on the distribution of effect modifiers
Source: BMC Med. 2013 Jul 4;11:159. doi: 10.1186/1741-7015-11-159 (PMC3707819; doi:10.1186/1741-7015-11-159)
Supplement: Additional file 1 — Equations to illustrate that the imbalance in the distribution of effect modifiers across different types of direct comparisons violates the consistency assumption of network meta-analysis and result in biased indirect estimates. [file 1741-7015-11-159-S1.docx]

**Imbalance in the distribution of an effect modifier across different types of direct comparisons violates the consistency assumption of network meta-analysis and result in biased indirect estimates**

Let us assume we can have randomized AB, AC, BC and ABC comparisons. We define$\delta_{ABj}$,$\delta_{ACj}$and $\delta_{BCj}$as the study specific true treatment effects of study *j* comparing intervention B with A, C with A, and C with B respectively. If we assume that between-study heterogeneity in treatment effects across all studies is only caused by a study-level effect modifier *x,* and there are no other sources of heterogeneity then:

$\begin{matrix} \delta_{ABj}=d_{AB}+\beta_{AB}x_{j} \\ \delta_{ACj}=d_{AC}+\beta_{AC}x_{j} \\ \delta_{BCj}=d_{BC}+\beta_{BC}x_{j} \end{matrix}$ (1)

where $d_{AB}$ $d_{AC}$ and $d_{BC}$are the treatment effects when the study level effect-modifier *x*=0. $x_{j}$represents the study level value of the effect modifier in study *j*. $\beta_{AB}$,$\beta_{AC}$and $\beta_{BC}$reflect the impact of the effect modifier on the study specific treatment effects. If$\beta_{AB}=\beta_{AC}=\beta_{BC}=0$ then variable *x* is not an effect modifier and there is no heterogeneity across studies.

In a three-arm randomized ABC study *j*, the relationship of the 3 treatment effects is defined as:

$\delta_{BCj}=\delta_{ACj}-\delta_{ABj}$ (2)

Combining (1) and (2) and we obtain:

$d_{BC}+\beta_{BC}x_{j}=d_{AC}+\beta_{AC}x_{j}-\left(d_{AB}+\beta_{AB}x_{j}\right)$ (3)

which can be reorganized according to:

$d_{BC}+\beta_{BC}x_{j}=d_{AC}-d_{AB}+\left(\beta_{AC}-\beta_{AB}\right)x_{j}$ (4)

From (4) it follows that

$d_{BC}=d_{AC}-d_{AB}$ (5)

and

$\beta_{BC}=\beta_{AC}-\beta_{AB}$ (6)

Equation (5) reflects the consistency assumption regarding the treatment effects when the covariate *x*=0. Equation (6) reflects the consistency assumption for the impact of the effect modifier on treatment effects for AB, AC and BC comparisons.

Given (2) the expected values for$\delta_{ABj}$,$\delta_{ACj}$and $\delta_{BCj}$are related according to:

$E\left(\delta_{BCj}\right)=E\left(\delta_{ACj}\right)-E\left(\delta_{ABj}\right)$ (7)

In combination with (4) we obtain:

$d_{BC}+\beta_{BC}E\left(x_{j}\right)=d_{AC}-d_{AB}+\left(\beta_{AC}-\beta_{AB}\right)E\left(x_{j}\right)$ (8)

which shows that the consistency equations (5) and (6) not only hold for a specific three arm ABC trial *j*, but also hold for a meta-analysis of several three arm ABC trials.

Now let us assume we have performed a meta-analysis of AB studies as well as a meta-analysis of AC studies and we want to obtain an indirect estimate for the BC comparison. According to (1) and (7) we have:

$E\left(\delta_{BCj}\right)=d_{AC}+\beta_{AC}E\left(x_{ACj}\right)-\left(d_{AB}+\beta_{AB}E\left(x_{ABj}\right)\right)=d_{AC}-d_{AB}+\beta_{AC}E\left(x_{ACj}\right)-\beta_{AB}E\left(x_{ABj}\right)$ (9)

It is obvious that equation (9) is equivalent to (8) when $E\left(x_{ABj}\right)=E\left(x_{ACj}\right)=E\left(x_{j}\right)$. In words: when the distribution of effect modifier *x* across AB and AC studies is similar then the indirect comparison of the result of a meta-analysis of AB studies with the result of a meta-analysis of AC studies gives a similar estimate for the BC comparison as would be obtained with a meta-analysis of ABC studies. If there is no imbalance in the distribution of the effect modifier between AB and AC studies then the indirect comparison is unbiased.

From (8) and (9) we can also infer that if $E\left(x_{ABj}\right)\neq E\left(x_{ACj}\right)$ then (8) and (9) are only equivalent when $\beta_{AC}=\beta_{AB}=0$. In words: in the presence of an imbalance in the distribution of a covariate between AB and AC studies, the indirect comparison is only valid when the covariate is not an effect-modifier.
